# Acute Paraspinal Compartment Syndrome in an Unconscious Patient

**DOI:** 10.7759/cureus.7216

**Published:** 2020-03-09

**Authors:** Talha Ahmed, Ayesha Safdar, Tamoor Ahmed, Talal Ahmad, Lia Losonczy

**Affiliations:** 1 Internal Medicine, University of Maryland Medical Center, Baltimore, USA; 2 Internal Medicine, Army Medical College, Rawalpindi, PAK; 3 Internal Medicine, King Edward Medical University/Mayo Hospital, Lahore, PAK; 4 Internal Medicine, Services Hospital, Lahore, PAK; 5 Emergency Medicine, The George Washington University Medical Center, Washington DC, USA

**Keywords:** compartment syndrome, paraspinal compartment syndrome, substance abuse, seizure, unconscious

## Abstract

Compartment syndrome can be a limb-threatening emergency that may require immediate intervention. It usually involves the extremities but any closed compartment of the body is susceptible to it. Paraspinal compartment extends on both sides of the spine. Prolonged lying on the back in unconscious patients leads to muscle edema which eventually leads to increase pressure in the compartment. Neurovascular comprise is a dreaded complication of compartment syndrome. Paraspinal compartment is a potential site of compartment syndrome particularly in unconscious patients and it requires prompt diagnosis, careful monitoring, immediate medical attention and even warranting surgical intervention in certain cases.

## Introduction

Compartment syndrome can be a limb-threatening emergency that may require immediate intervention. It can involve any closed compartment of the body with the most common site being the anterior distal lower extremity or the anterior compartment of the leg [1]. We describe a case of paraspinal compartment syndrome in an unconscious patient, secondary to polysubstance abuse and seizures. Prolonged periods of pressure on the compartments of the back particularly the paraspinal compartment can lead to paraspinal compartment syndrome which can be easily missed in an unconscious patient. This requires performing a thorough clinical examination particularly in patients with unexplained elevations in creatine kinase (CK) levels that are not being cleared from the body. Appropriate and timely intervention can prove to be life saving [2].

## Case presentation

**Figure 1:**
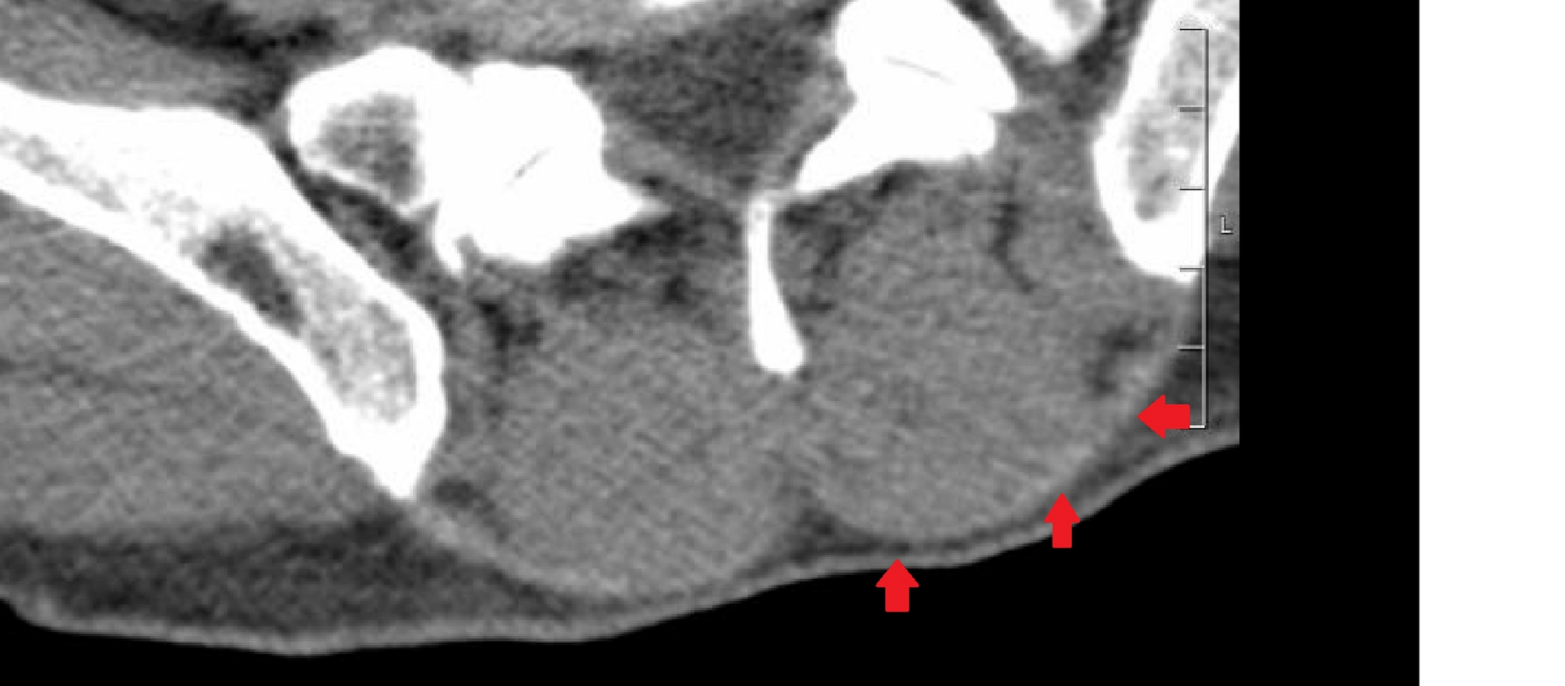
Computed tomography scan of the lumbar spine showing swelling of the left paraspinal compartment

A 49-year-old male with a past history of seizures on antiepileptics was brought to the hospital (due to confusion and altered mental status) after his wife called the emergency medical service (EMS). The patient had a reported episode of seizure on arrival to the EMS after which he continued to remain confused and somnolent. The patient had stable vital signs on presentation with a heart rate of 65 beats per minute, blood pressure of 130/78 mmHg, a body mass index (BMI) of 26 kg/m2 and was afebrile with oxygen saturation of 95% on room air. Urine toxicology screen in the emergency department (ED) was positive for cocaine and the urine fentanyl dip test was positive. Computed tomography (CT) of the head was normal. Creatine kinase (CK) was elevated to 4550 units/L (normal range: 22-198 units/L) at the time of admission. Other significant lab findings included mild transaminitis with aspartate aminotransferase (AST) of 148 units/L (normal range: 10-40 units/L) and alanine aminotransferase (ALT) of 90 units/L (normal range: 7-56 units/L). The patient was admitted to the intensive care unit (ICU), was given a bolus of three liters fluids, and continued on maintenance fluids to produce 200 ml of urine per hour. Serial measurements of CK did not show significant improvement despite fluid resuscitation and stayed at 4530 units/L even after 24 hours of significant hydration. This prompted a complete re-examination of the body to evaluate for compartment syndrome which revealed significant swelling on the left paraspinal area in the lower thoracic and lumbar region extending laterally along the external oblique muscle. As the illicit drugs started clearing from the patient's body, his mental status improved and he was able to complain of pain and tenderness to palpation at the swollen site. CT scan of the abdomen and pelvis, as well as the lumbar and thoracic spine, were ordered for evaluation of the swelling. There was asymmetry and edema along the left paraspinal muscles especially the external oblique (Figure 1).

The patient was re-positioned to decrease compression on the affected site while continuing fluid resuscitation and general surgery was consulted for possible intervention, if needed. It was decided to do conservative management initially with frequent clinical examinations, serial CK level monitoring. and re-positioning of the patient. Repeat CK after 36 hours of being in the ICU started trending down with the left-sided paraspinal area showing decreased swelling, pain, and tenderness; hence, it did not warrant measuring the tissue pressures. Fortunately, the patient’s renal function remained intact.

## Discussion

Paraspinal compartment syndrome is a rare entity that requires a high index of suspicion for diagnosis. This syndrome can be classified into acute or chronic types. Acute paraspinal compartment syndrome subtypes include the following: atraumatic (due to downhill skiing, surf boarding, weight lifting), direct trauma (accidents, prolonged pressure, or direct injury) and secondary to non-spinal surgery (due to aortic bypass, gastric bypass) [3]. This patient presented with acute paraspinal compartment syndrome from prolonged pressure on the affected compartment from unconsciously lying down on the back for a long period of time due to substance abuse and seizures. 

Unlike extremity compartment syndrome where classical signs include pain, paresthesias, paresis, pallor, poikilothermia, and pulselessness, in acute paraspinal compartment syndrome, all these symptoms are not appreciated [4]. Typical clinical presentation includes localized pain, tenderness, rigidity, and paresthesia [5]. Interestingly, patients may also have decreased or absent bowel sounds and activities that raise the intra-abdominal pressure which may exacerbate symptoms [6].

Laboratory findings supporting the diagnosis include elevated CK, urine myoglobin, creatinine, AST, and ALT [7]. Edema, inflammation, and/or hematoma on magnetic resonance imaging (MRI) usually identifies the paraspinal compartment [8,9]. Measuring of intra-compartment pressures confirms the diagnosis with pressures that are usually significantly elevated (70-80 mmHg) [10].

Once acute paraspinal compartment syndrome is suspected, management should focus on the following: aggressive resuscitation with intravenous fluids, avoidance of nephrotoxic drugs, cessation of any further traumatic activities, and urgent surgical decompression with fasciotomy. Although conservative management with non-operative intervention is an option, patients have demonstrated improved outcomes with surgical fasciotomy even when it is delayed [11].

In our case, conservative management with very close monitoring and aggressive resuscitation yielded favorable outcomes. This case, however, demonstrates that in unconscious patients with persistently elevated CK, we should be extremely vigilant with our clinical examination to evaluate all the compartments of the body particularly the paraspinal compartment. This article was presented as a poster. (Poster: Talha Ahmed, Ayesha Safdar, Lia Losonczy. Acute Paraspinal Compartment Syndrome Secondary to Polysubstance Abuse and Seizures. CHEST Annual Meeting; Oct 2019).

## Conclusions

Paraspinal compartment syndrome can be acute secondary to weight lifting, skiing, surfing, direct trauma, non-spinal surgery including aortic, and gastric bypass or chronic secondary to chronic exertion. Conservative measures are the first line of management and include opioids for pain control and treatment of rhabdomyolysis. Although rare, it is prudent to consider the paraspinal area as a potential site for acute compartment syndrome in unconscious patients with a history of substance abuse and significantly elevated CK. By providing appropriate interventions, potentially serious consequences can be avoided.

## Appendices

The abstract of this case report was presented as a poster at the 'CHEST' conference in 2019. 
